# The immatures of *Bezziachilensis* Spinelli & Ronderos, 2001 (Diptera, Ceratopogonidae)

**DOI:** 10.3897/zookeys.803.29024

**Published:** 2018-12-06

**Authors:** Danielle Anjos-Santos, Florentina Díaz, Gustavo Ricardo Spinelli, María Marcela R nderos

**Affiliations:** 1 Laboratório de Investigaciones en Ecología y Sistemática Animal, CIEMEP, UNPSJB, CONICET-CCT-PATAGONIA NORTE, Gral. Roca 780, Esquel (9200), Chubut, Argentina Laboratório de Investigaciones en Ecología y Sistemática Animal Esquel Argentina; 2 División Entomología, Museo de La Plata, UNLP-FCNYM, Paseo del Bosque s/n, La Plata (1900), Buenos Aires, Argentina División Entomología, Museo de La Plata La Plata Argentina; 3 Centro de Estudios Parasitológicos y de Vectores (CEPAVE), CONICET, Boulevard 120 s/n e/61 y 62, La Plata (1900), Buenos Aires, Argentina Centro de Estudios Parasitológicos y de Vectores La Plata Argentina; 4 Instituto de Limnología “Dr. Raúl A. Ringuelet” (ILPLA), CONICET, Boulevard 120 s/n e/Avda. 60 y calle 64, La Plata (1900), Buenos Aires, Argentina Instituto de Limnología “Dr. Raúl A. Ringuelet” La Plata Argentina

**Keywords:** Aquatic, biting midges, immature stages, Neotropical region, Palpomyiini

## Abstract

The fourth instar larva and the pupa of *Bezziachilensis* Spinelli & Ronderos, 2001 are described for the first time. The immature stages were collected from macrophytes and filamentous algae in streams of the Patagonian steppe, in the provinces of Neuquén and Chubut, Argentina. The described stages were photographed and illustrated with a phase-contrast microscope and scanning electron microscope. Data on the bionomics of the species, new records and tables for characters of the known larvae and pupae of *Bezzia* Kieffer, 1899 from the Neotropical region are provided.

## Introduction

*Bezzia* Kieffer, 1899, a worldwide genus of the tribe Palpomyiini, includes 322 species of which 48 inhabit the Neotropical region, 46 of them recorded by Borkent and Spinelli (2007), and two more recently described: *B.ventanensis* Spinelli, 2012 (Spinelli et al. 2012) and *B.galesa* Spinelli, 2013 (Spinelli et al. 2013). The adults are important predators of small invertebrates and the immature stages are relatively common inhabitants of various kinds of freshwater environments, mainly streams, lakes and ponds, as well as other breeding habitats, such as sphagnum bogs, rice fields, footprints in sandy creek beds, and water gathered in tree holes and bromeliads (Spinelli and Ronderos 2001). The majority of the Neotropical species are known from adults, and only 12 of them are also known as immatures: *B.bivittata* (Coquillett, 1905), *B.blantoni* Spinelli & Wirth, 1989, *B.brevicornis* (Kieffer, 1917); *B.bromeliae* Spinelli, 1991; *B.galesa* Spinelli, 2013; *B.gibbera* (Coquillett, 1905); *B.glabra* (Coquillett, 1902), *B.nobilis* (Winnertz, 1852), *B.pulchripes* Kieffer, 1917; *B.roldani* Spinelli & Wirth, 1981, *B.snowi* Lane, 1958; and *B.ventanensis* Spinelli, 2012.

*Bezziachilensis* Spinelli & Ronderos, 2001 is a member of the *venustula* species group in the subgenus Homobezzia Macfie, 1932, distributed in Valparaiso Province (Chile), and Salta and Río Negro provinces (Argentina) (Spinelli and Cazorla 2003). During a recent survey carried out in the northwestern Argentine Patagonia, larvae and pupae of *B.chilensis* were collected. The purpose of this paper is to describe the fourth instar larva and pupa of this species, with phase-contrast and scanning electron microscopy (SEM) and to provide tables for characters of the known larvae and pupae of *Bezzia* from the Neotropical region.

## Material and methods

Larvae and pupae were collected on the bordering vegetation in three streams on the Patagonian steppe in the provinces of Neuquén and Chubut. The substrate was removed with the aid of a strainer and transferred to a white tray where larvae and pupae were collected with a pipette. Further substrate samples were carried to the laboratory to search for more specimens. Larvae were placed in individual containers with water and substrate from their natural environment. Pupae were isolated in a vial with a drop of water, and observed daily until adult emergence. Adults were allowed to harden for 24 h before being preserved in ethanol to ensure their complete pigmentation. For detailed examination with a phase-contrast microscope, larval and pupal exuviae and adults were mounted in Canada balsam following the technique described by Borkent and Spinelli (2007). Mounted larval exuviae were oriented ventral side up to facilitate examination of the epipharyngeal combs within the head capsule. Pupal exuviae were mounted dorsoventrally. Photomicrographs were taken with a Micrometrics SE Premium digital camera, through a Nikon Eclipse E200 microscope and a Leica EC3 digital camera, through a Leica DM 500 microscope. Illustrations were drawn with a camera lucida and Adobe illustrator CC. The map was drawn in QGIS v. 2.14. Larvae were also examined using scanning electron microscopy (SEM) (JOEL 2000) following the technique of Ronderos et al. (2000, 2008). Measurements were taken with a (BCM) Leitz Wetzlar binocular compound microscope. The temperature of the water and air were measured with an alcohol thermometer in degrees Celsius. For larval terms and abbreviations of measurements, see Anjos-Santos et al. (2017); for pupal terms, see Borkent (2014). Studied specimens are deposited in the collection of the Museo de La Plata, La Plata, Argentina (MLPA).

## Results

### Bezzia chilensis

Taxon classificationAnimaliaDipteraCeratopogonidae

Spinelli & Ronderos, 2001

**Figure 1.**
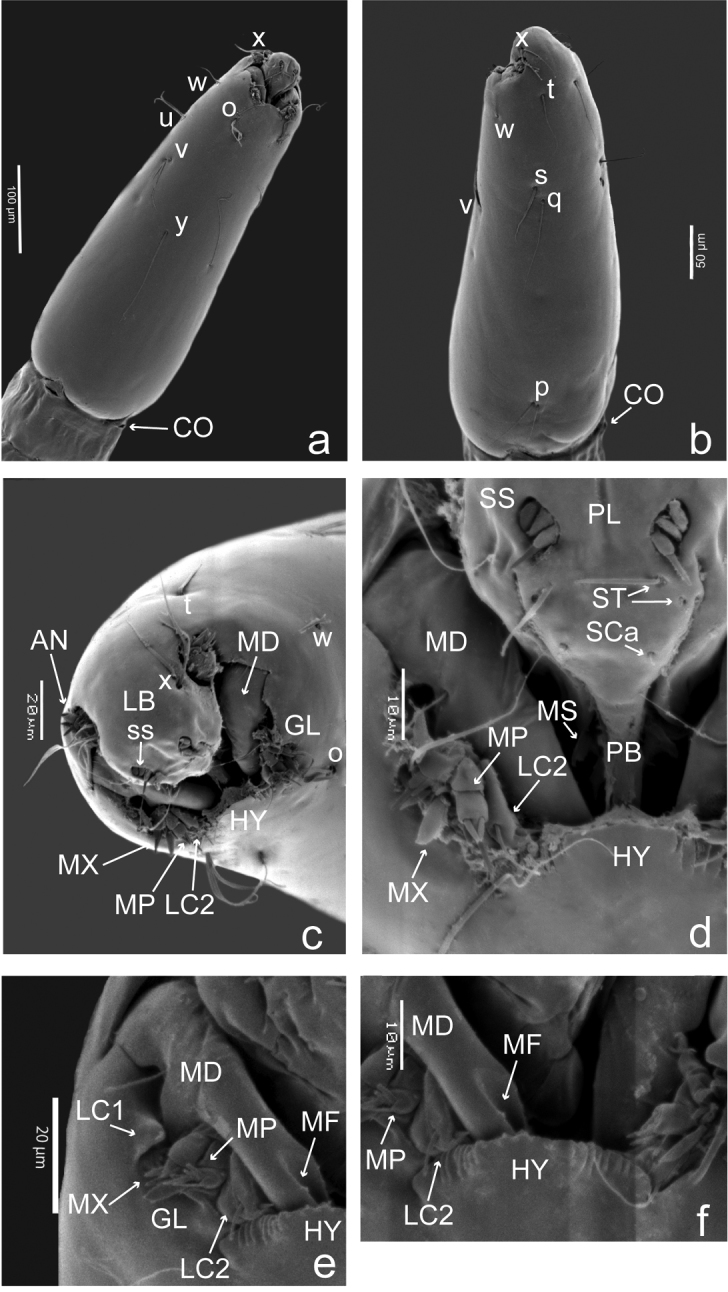
*Bezziachilensis* Spinelli & Ronderos, 2001, fourth instar larva (SEM) **a** Head chaetotaxy, ventrolateral view **b** Head chaetotaxy, dorsolateral view **c** Head capsule detail, oblique anteroventral view **d** Head capsule detail, ventral view **e** Mouthpart, ventral view **f** Hypostoma and mouthparts, ventral view. Antennae (AN); collar (CO); fossa mandibularis (MF); galeolacinea (GL); hypostoma (HY); labrum (LB); lacinial sclerite 1 (LC1); lacinial sclerite 2 (LC2); mandible (MD); messors (MS); maxilla (MX); maxillary palpus (MP); palatal bar (PB); palatum (PL); sensilla campaniformia (SCa); sensilla styloconica (SS); sensilla trichoidea (ST); Head capsule chaetotaxy: o, parahypostomal setae; p, posterior perifrontal setae; q, postfrontal setae; s, anteroperifrontal setae; t, prefrontal setae; u, mesolateral setae; v, posterolateral setae; w, anterolateral setae; x, parantennal setae; y, ventral setae.

**Figure 2.**
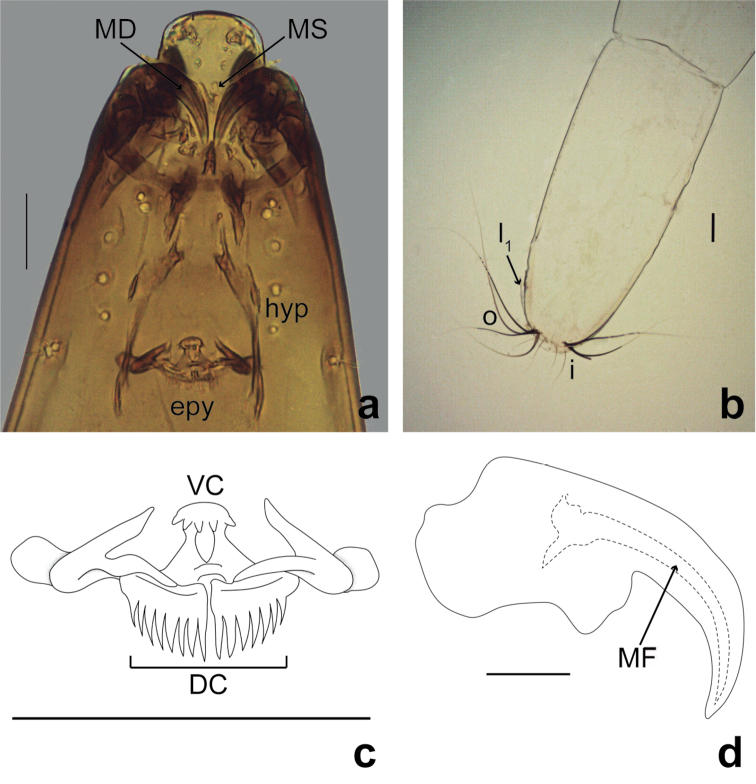
*Bezziachilensis* Spinelli & Ronderos, 2001, fourth instar larva **a** Head capsule detail, ventral view **b** Caudal segment, ventral view **c** Epipharynx, ventral view **d** Left mandible, ventral view. Dorsal comb (DC); epipharynx (epy); fossa mandibularis (MF); hypopharynx (hyp); mandible (MD); messors (MS); ventral comb (VC). Caudal segment chaetotaxy: i, inner setae; l_1_, first lateral seta; o, outer setae. Scale bars: 0.05 mm (**a–c**), 0.01 mm (**d**).

**Figure 3.**
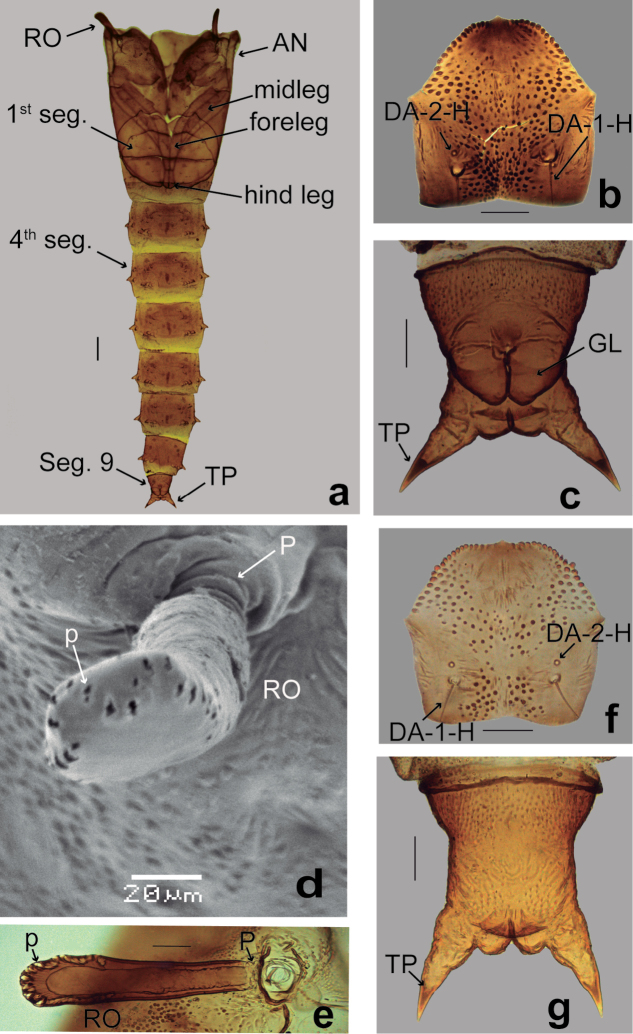
*Bezziachilensis* Spinelli & Ronderos, 2001, male pupa (**a–d**), female pupa (**e–g**) **a** Habitus, ventral view **b, f** Dorsal apotome, dorsal view **c, g** Segment 9, ventral view **d** Respiratory organ, anterodorsal view (SEM) **e** Respiratory organ ventral view. Antenna (AN); dorsal apotome sensilla (DA-1-H, DA-2-H); genital lobe (GL); pore (p); pedicel (P); respiratory organ (MD); segment 1 (1^st^ seg.); segment 4 (4^th^ seg.); segment 9 (Seg. 9); terminal process (TP). Scale bar: 0.05 mm.

**Figure 4.**
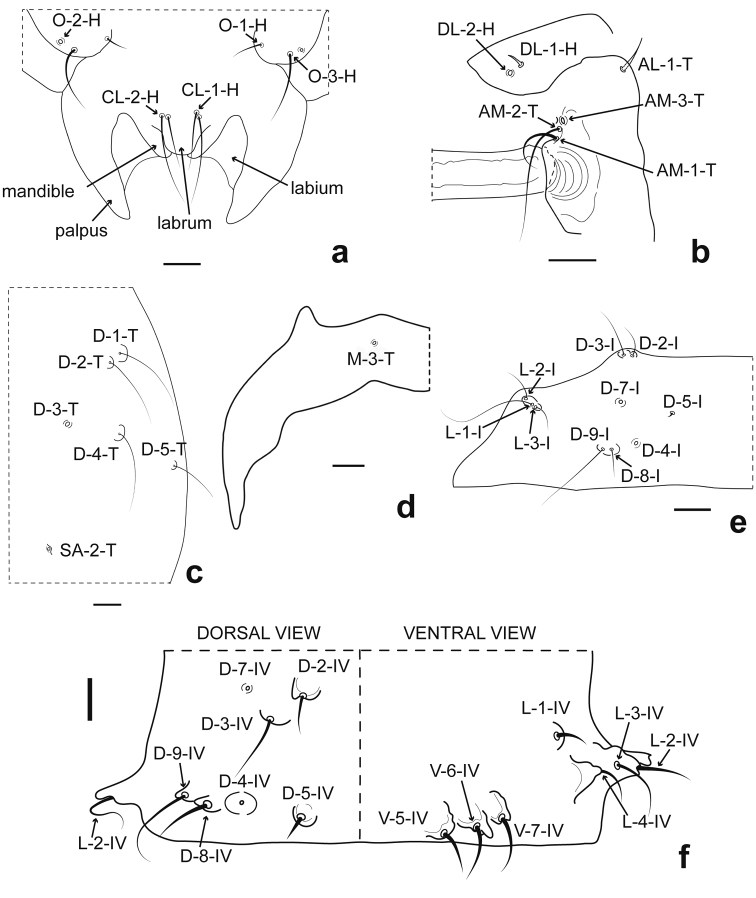
*Bezziachilensis* Spinelli & Ronderos, 2001, female pupa **a** Mouthparts, ventral view **b** Anterolateral, anteromedial and dorsal lateral sensilla, ventral view **c** Dorsal sensilla and supraalar sensillum, dorsal view **d** Metathoracics chaetotaxy, dorsal view **e** Tergite 1 chaetotaxy, dorsal view **f** Segment 4 chaetotaxy, dorsal and ventral view. Anterolateral sensillum (AL-1-T); anteromedial sensilla (AM-1-T, AM-2-T, AM-3-T) ; clypeal/labral sensilla (CL-1-H, CL-2-H); dorsal sensilla (D-1-T, D-2-T, D-3-T, D-4-T, D-5-T); dorsal sensilla of segment 1 (D-2-I, D-3-I, D-4-I, D-5-I, D-7-I, D-8-I, D-9-I); dorsal sensilla of segment 4 (D-2-IV, D-3-I, D-4-I, D-5-IV, D-7-IV, D-8-IV, D-9-IV); dorsolateral cephalic sclerite sensilla (DL-1-H, DL-2-H); lateral sensilla of segment 1 (L-1-I, L-2-I, L-3-I); lateral sensilla of segment 4 (L-1-IV, L-2-IV, L-3-IV, L-4-IV); ocular sensilla (O-1-H, O-2-H, O-3-H); metathoracic sensillum (M-3-T); supraalar sensillum (SA-2-T); ventral sensilla of segment 4 (V-5-IV, V-6-IV, V-7-IV). Scale bar: 0.05 mm.

Figs 1a–f , 2a–d , 3a–g , 4a–f

Bezzia chilensis : Spinelli and Ronderos 2001: 752 (male, female; Chile); Spinelli and Cazorla 2003: 47 (Argentina records); Borkent and Spinelli 2007: 93 (in Neotropical catalogue); Spinelli and Marino 2009: 205 (species list from Patagonia); Borkent 2016: 160 (in online world catalog).

#### Description of fourth instar larva

(Figs 1a–f, 2a–d). Head capsule (Figs 1a–c, 2a) pale brown, about 2 times longer than wide, apex slightly bent ventrally, HL 0.30–0.32 (0.31, *n* = 7) mm; HW 0.12–0.19 (0.16, *n* = 7) mm, HR 1.70–2.41 (2.00, *n* = 6); SGW 0.083–0.116 (0.098, *n* = 6) mm; SGR 1.29–1.82 (1.66, *n* = 6). Setae simple, thin, medium to long sized, chaetotaxy as in Figure 1a–c. Antenna bottom-shaped, small, length 0.01 (*n* = 3) mm. Labrum (Fig. 1c) longer than wide, not extending beyond hypostoma, with three pairs of anterolateral sensilla styloconica; palatum (Fig. 1d) with two pairs of closely spaced sensilla, one trichoidea, one campaniform sensillum; messors (Figs 1d, 2a) small, gently sclerotized, curved structures, situated away from mandibles, without scopae; palatal bar present (Fig. 1d), triangular, situated immediately posterior to messors. Mandible (Figs 1c–f, 2a, d) hooked, curved, strongly sclerotized, apical tooth long, deep fossa mandibularis on ectal surface; MDL 0.045–0.073 (0.052, *n* = 6) mm, MDW 0.02 (*n* = 6) mm. Maxilla (Fig. 1c–e) with pyriform sensillum, galeolacinia with lacinial sclerite 1 (Fig. 1e) and lacinial sclerite 2 (Fig. 1c–f) with 2 setae, one medium-sized stout, other short; maxillary palpus (Fig. 1c–f) cylindrical, with 4 subapical papillae, three medium-sized, one elongate. Hypostoma (Fig. 1c–f) finely toothed, with 6 or 7 stout lateral teeth. Epipharynx (Fig. 2a, c) less massive, with 2 combs: ventral comb with 5 stout, short teeth, dorsal comb with 7 or 8 long, pointed teeth on posterior edge; lateral arms elongate; LAW 0.048–0.050 (0.049, *n* = 4) mm, DCW 0.025–0.038 (0.028, *n* = 4) mm. Hypopharynx (Fig. 2a) elongate, thin, gently sclerotized, arms slender, without fringe. Thoracic pigmentation uniformly pale yellowish. Caudal segment (Fig. 2b) about 2.7 times as long as wide, with one pair of long, stout setae “o”, one medium-sized, thin setae “i”, one pair of short, thin setae “l_1_”. CSL 0.51–0.57 (0.55, *n* = 5) mm, CSW 0.21–0.28 (0.25, *n* = 5) mm, CSR 1.88–2.71 (2.7, *n* = 5), OL 0.20–0.30 (0.27, *n* = 6) mm, OD 0.020–0.072 (0.049, *n* = 5) mm.

#### Description of pupa.

**Female** (Figs 3e–g, 4a–f). Habitus as male pupa (Fig. 3a). Exuviae brownish. Total length 3.14–3.91 (3.48, *n* = 13) mm. *Head*: Dorsal apotome (Fig. 3f) with disc surface bearing rounded small tubercle mesally, anterior margin slightly rounded, covered with stout, rounded spinules; posterior margin slightly concave, mesal portion with pair of raised areas; antenna extending posteriorly to midleg; mouthparts (Fig. 4a) with mandible well developed; palpus extending to posterolateral margin of labium; labium separated medially by labrum; apex of labrum slightly rounded; sensilla: dorsal apotomals (Fig. 3f): DA-1-H elongate, stout seta, located on rounded small tubercle, DA-2-H campaniform sensillum; DAL 0.08–0.10 (0.09, *n* = 10) mm; DAW 0.20–0.23 (0.22, *n* = 10) mm; DAW/DAL 2.24–2.64 (2.46, *n* = 10); two dorsolateral cephalic sclerites (Fig. 4b): DL-1-H short, stout seta, DL-2-H campaniform sensillum; clypeal/labrals (Fig. 4a): CL-1-H medium-sized thin seta CL-2-H long, thin seta; oculars (Fig. 4a): O-1-H short, stout seta, O-2-H campaniform sensillum, O-3-H long, stout seta. Cephalothorax rectangular, surface predominantly smooth with small spinules on mesonotum, between bases of respiratory organs. Length of cephalothorax 1.12–1.35 (1.22, n=13) mm, width 0.81–1.00 (0.92, *n* = 13) mm. *Thorax*: Respiratory organ (Fig. 3d, e) smooth, medium-sized, pale brown except distal 1/3 darker, about 3.05–5.70 (4.38, *n* = 13) times longer than broad, almost straight with rounded apex, with convoluted row of 30–35 (33, *n* = 13) pores closely abutting at apex and apicolateral 1/4 of respiratory organ; pedicel slender, P 0.024–0.040 (0.034, *n* = 13) mm; RO length 0.20–0.24 (0.22, *n* = 13) mm, RO width 0.04–0.08 (0.05, *n* = 13) mm; P/RO 0.10–0.18 (0.15, n=13); sensilla: three anteromedials (Fig. 4b): AM-1-T medium-sized, stout seta, AM-2-T long, thin seta, AM-3-T campaniform sensillum; one anterolateral (Fig. 4b): AL-1-T medium-sized, stout seta; dorsals (Fig. 4c): D-1-T, D-2-T, D-4-T, long, thin setae, D-3-T campaniform sensillum, D-5-T medium-sized, thin seta, all on small rounded tubercle; supraalar (SA-2-T) campaniform sensillum; metathoracic (Fig. 4d): M-3-T campaniform sensillum, near anterior margin of metathorax. *Abdomen*: Abdominal segments with dark spots, with simple setae, covered with very small spicules, segment 9 (Fig. 3g) approximately twice as long as wide, length 0.24–0.29 (0.27, *n* = 13) mm, width 0.14–0.20 (0.17, *n* = 13) mm; dorsal surface covered with pointed spicules; ventral surface smooth; terminal process moderately short, nearly straight, base wide, smooth, extreme tips darker, length 0.07–0.10 (0.09, *n* = 13) mm, width 0.03–0.04 (0.04, *n* = 13) mm; sensilla: tergite 1 (Fig. 4e) with two anteromesals: D-2-I medium-sized, thin seta, D-3-I long, thin seta; 5 posterior sensilla: D-4-I, D-7-I campaniform sensilla, D-5-I minute seta, D-8-I medium-sized, thin seta, D-9-I long, thin seta; 3 lateral sensilla: L-1-I long, thin seta, L-2-I, L-3-I medium-sized, thin setae; segment 4 (Fig. 4f): D-2-IV medium-sized, thin seta, D-3-IV long, thin seta, D-4-IV, D-7-IV campaniform sensilla, D-5-IV short, stout seta, D-8-IV medium-sized, stout seta, D-9-IV long, thin seta; L-1-IV short, stout seta, L-2-IV long, thin seta, L-3-IV, L-4-IV medium-sized, stout setae, all on bifid tubercles with wide base and pointed apex; V-5-IV, V-6-IV, V-7-IV medium-sized, stout setae, all on elongate tubercles; segment 9 (Fig. 3g) with D-5-IX campaniform sensillum.

**Male** (Fig. 3a–d). Similar to female with usual sexual differences: Total length 2.68–3.77 (*n* = 15) mm. Dorsal apotome (Fig. 3b) darker with anterior margin slightly triangular, DAL 0.07–0.10 (0.08, *n* = 12) mm; DAW 0.18–0.22 (0.19, *n* = 12) mm, DAW/DAL 2.0–3.0 (2.4, *n* = 12). Cephalothorax: length 0.97–1.17 (1.09, *n* = 15) mm, width 0.70–0.85 (0.77, *n* = 13) mm. Respiratory organ (Fig. 3d), about 3.36–4.90 (4.01, *n* = 15) times longer than broad, P 0.020–0.036 (0.031, *n* = 15) mm; RO length 0.168–0.244 (0.205, *n* = 15) mm, RO width 0.040–0.060 (0.051, *n* = 15) mm; P/RO 0.106–0.196 (0.156, *n* = 15). Segment 9 (Figs 3a, c) darker, ventral surface covered anteriorly with pointed spicules, length 0.124–0.288 (0.241, *n* = 15) mm, width 0.120–0.248 (0.153, *n* = 15) mm; terminal process length 0.07–1.00 (0.08, *n* = 15) mm, width 0.032–0.044 (0.037, *n* = 15) mm; genital lobe short, each slightly longer than wide and apex anterior to base of terminal process.

#### Material examined.

Argentina, Neuquén Province, Parque Nacional Nahuel Huapi, río Cuyín Manzano, 40°44'13"S, 71°09'17"W, alt. 760 m, 06-II-2009, A. Siri, 2 females and 1 male (with pupal exuviae). Argentina, Chubut Province, Ruta Nacional 40, arroyo La Cancha, 42°45'35.9"S, 71°06'28.4"W, alt. 860 m, 13-II-2015, adults emerged in laboratory 14-II-2015, D. Anjos-Santos and P. Pessacq, 1 female, 3 males (with pupal exuviae); same data except adult emerged 15-II-2015, 1 male (with pupal exuviae); same data except adults emerged 17-II-2015, 2 females, 1 male (with pupal exuviae); same data except adults emerged 18-II-2015, 2 females, 1 male (with pupal exuviae); same data except adults emerged 17-II-2015, 1 female, 1 male (with pupal exuviae); same data except pupa emerged in laboratory 26-II-2015, adult emerged 28-II-2015, 1 female (with larval and pupal exuviae); same data except adult emerged 03-III-2015, 1 female (with pupal exuviae); Argentina, Chubut prov., Ruta Nacional 40, arroyo Madera, 42°39'57.59"S, 71°04'19.72"W, alt 930 m, 18-II-2015, pupa emerged in laboratory 23-II-2015, adult emerged 28-II-2015, D. Anjos-Santos and P. Pessacq, 1 male (with larval and pupal exuviae); same data except pupae emerged 23-II-2015, adults emerged 02-III-2015, 2 females (with larval and pupal exuviae); same data except pupa emerged 24-II-2015, 1 male (with pupal exuviae); same data except pupa emerged 26-II-2015, 1 male (with pupal exuviae); Argentina, Chubut Province, arroyo Montoso, 42°42'01.26"S; 70°48'12.36"W, alt. 630 m, 14-I-2016, pupa emerged in laboratory 16-I-2016, D. Anjos-Santos and P. Pessacq, 1 male (with pupal exuviae); same data except pupa emerged 17-I-2016, adult emerged 21-I-2016, 1 female (with larval and pupal exuviae); same data except pupa emerged 17-I-2016, adult emerged 22-I-2016, 1 male (with larval and pupal exuviae); same data except pupa emerged 20-I-2016, adult emerged 25-I-2016, 1 male (with larval and pupal exuviae).

#### Material examined by SEM.

Argentina, Chubut Province, Ruta Nacional 40, arroyo Madera, 42°39'57.59"S, 71°04'19.72"W, alt. 930 m, 18-II-2015, D. Anjos-Santos and P. Pessacq, 3 larvae, 1 male pupa.

#### Distribution.

Argentina (Salta, Neuquén, Río Negro and Chubut provinces); Chile (Valparaiso Province).

## Bionomics

**Figure 5.**
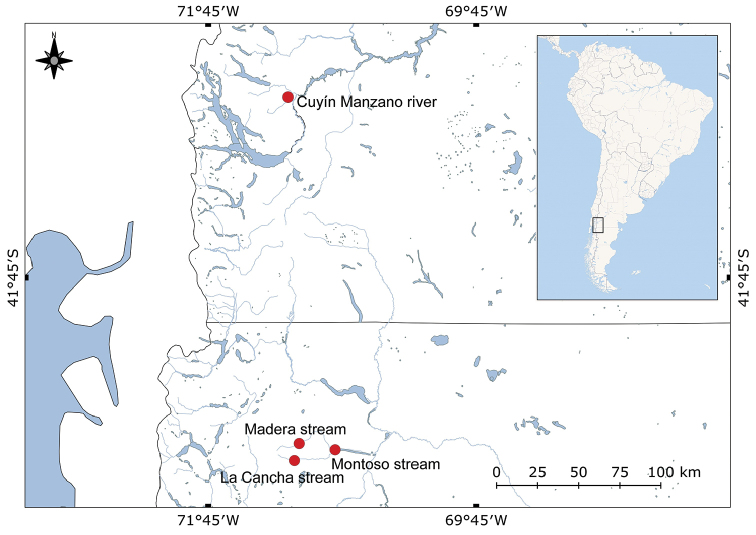
Collection sites of *Bezziachilensis* Spinelli & Ronderos, 2001.

The immature described here were collected in northwestern Argentine Patagonian steppe (Fig. 5): río Cuyín Manzano, located in the Parque Nacional Nahuel Huapi in southern Neuquén Province and arroyo La Cancha, arroyo Madera and arroyo Montoso, that flow into tributaries of the Chubut river in northern Chubut Province. All sites are surrounded by shrubby steppe, composed mainly of willow tree (Salicaceae) and grass, and are used as a water source for cattle. Immatures were collected in puddles of water with a rocky or sandy bottom, on the bank of the streams, among macrophytes (*Ceratophyllum* L.), bryophytes and filamentous algae. Larvae were distributed through all the puddles, but pupae only in the bordering vegetation. In La Cancha, Madera and Montoso streams water temperature ranged between 15–19 °C, and air temperature between 16–25 °C. In Cuyín Manzano river, the air and water temperature data were not measured. Under laboratory conditions, the larvae took 4–14 days to reach the pupal stage, and 2–7 days to complete its development to the adult stage. Pupae found at the site completed their development in 1–8 days. Larvae of *Bezziachilensis* showed the same movement reported by Spinelli et al. (2013) for *B.galesa*, alternated fast undulating movements with static periods. Pupae observed on trays showed a semi-circular, slow abdominal movement typical of other ceratopogonid pupae.

## Taxonomic discussion

In a series of contributions reviewing the Neotropical *Bezzia*, Spinelli and Wirth (1989a, b, 1990, 1991) recognized the subgenus Bezzia, including the *gibbera*, *nobilis* and *punctipennis* groups, and the subgenus Homobezzia including the *dentifemur*, *glabra*, *venustula* and *brevicornis* groups. These papers also present diagnoses, descriptions and keys to subgenera and species groups, the last ones based mainly on adult characters. Spinelli et al. (2012) suggested a cladistic analysis is needed to propose a phylogenetic classification of the genus *Bezzia*.

Borkent (2014) presented a generic pupal description of *Bezzia* and in his taxonomic discussion mentioned the difficulties in diagnosing the genus and affirmed that providing a key to the species in a given region is superfluous. The current knowledge of immature stages of the Neotropical Bezzia is incipient. The subgenus Bezzia has only two species known as larvae and two known as pupae and the subgenus *Homobezzia* has five known as larvae and 10 known as pupae, with some of these immatures being poorly described and impossible to compare with their congenerics. Main diagnostic characters for larvae and pupae are given in the Tables 1 and 2, respectively.

The immatures of *Bezziachilensis* are herein compared with four species belonging to the subgenus Homobezzia; these four are the only ones which have a complete description: *B.blantoni* (described by Ronderos and Spinelli 2009), *B.galesa* (described by Spinelli et al. 2013), *B.roldani* (described by Ronderos et al. 2007) and *B.ventanensis* (described by Spinelli et al. 2012).

The larva of *Bezziachilensis* shares with *B.blantoni*, *B.galesa* and *B.roldani* features typical of predatory larvae: hooked mandibles with fossa mandibularis, epipharynx less massive with 2 combs and cylindrical maxillary palpus (Hribar and Mullen 1991). The labrum and palatum sensilla are very similar among the species. However, the larvae of these three species can be distinguished from *B.chilensis* by the features given in Table 1 and by the following additional characters: *Bezziablantoni* by the maxilla with a blunt sensillum, epipharynx with 4–6 stout, short teeth and auxiliary sclerite shorter; *Bezziagalesa* by the maxilla with a blunt sensillum, galeolacinia with a stout, sharp, pointed and medium-sized seta, epipharynx with 6 or 7 stout and small teeth on the ventral comb; and *Bezziaroldani* by the W-shaped palatal bar and the ventral comb of the epipharynx bearing 4 or 5 stout and short teeth. The larva of *B.ventanensis* remains unknown.

**Table 1.** Main diagnostic characters for the known larvae of Neotropical species of *Bezzia*.

Subgenus	species	Head capsule ratio	Head capsule setae	Hypostoma	Fossa mandibularis	Scopae	Maxillary palpus	Hypopharyngeal fringe	Caudal segment	Reference
* Bezzia *	* bivittata *	?	?	Finely toothed	Deep	Absent	3 subapical papillae	Absent	?	Hribar and Mullen (1991)
	* nobilis *	2.7 times as long as wide	Medium-sized to long	Finely toothed	Deep	Absent	?	Absent	2.5 times as long as wide	Wirth (1983b)
* Homobezzia *	* blantoni *	2.6–3.4 times as long as wide	Minute	Finely toothed, not flanked by stout teeth	Shallow	Present, with 5 teeth	2–3 subapical papillae	Present	4 times as long as wide	Ronderos and Spinelli (2009)
	* chilensis *	2 times as long as wide	Medium-sized to long	Finely toothed flanked by 6–8 stout teeth	Deep	Absent	4 subapical papillae	Absent	2 times as long as wide	This study
	* galesa *	2.8 times as long as wide	Minute	Finely toothed, with strong lateral teeth	Deep	Absent	4 subapical papillae	Absent	5–6 times as long as wide	Spinelli et al. (2013)
	* glabra *	4 times as long as wide	?	?	?	?	?	?	10 times as long as wide	Wirth (1983a)
	* roldani *	3.4 times as long as wide	Minute	Finely toothed, not flanked by stout teeth	Shallow	Absent	2–3 subapical papillae	Absent	5 times as long as wide	Ronderos et al. (2007)

With regard to the pupa, besides the features given in Table 2, these four species can be distinguished from *B.chilensis* as follows: *B.blantoni*: O-2-H absent, AL-2-T present; *B.galesa*: D-5-T, D-7-I and D-7-IV absent; *Bezziaroldani* O-2-H and V-5-IV absent; *Bezziaventanensis* AM-3-T absent.

**Table 2.** Main diagnostic characters for the known pupae of Neotropical species of *Bezzia*.

Subgenus	Species	Dorsal apotomals sensilla	Dorsolateral cephalic sclerite sensilla	Clypeal/labral sensilla	Oculars sensilla	Respiratory organ apex	Respiratory organ pores	Sensillum D-7-IV	Terminal process of segment 9	References
* Bezzia *	* nobilis *	1 seta, 2 campaniform	3 setae	2 setae	1 seta, 1 campaniform	Rounded	16–25 on distal 1/4	Present	Short	Borkent (2014); Wirth (1983b)
	* gibbera *	1 seta, 1 campaniform	?	?	?	?	?	?	?	Borkent (2014)
* Homobezzia *	* blantoni *	1 seta, 2 campaniform	1 seta	2 setae	2 setae	Rounded	31–35 on distal apex	Absent	Long	Ronderos and Spinelli (2009); Spinelli and Wirth (1989)
	* brevicornis *	1 seta, 1 campaniform	?	?	?	Asymmetrical (outer side curved, inner side straight)	23–25 on distal 1/3	?	Short	Spinelli and Wirth (1989)
	* bromeliae *	1 seta	?	?	?	Asymmetrical (outer side curved, inner side straight)	4 on distal apex	?	Short	Spinelli and Wirth (1991)
	* chilensis *	1 seta, 1 campaniform	1 seta, 1 campaniform	2 setae	2 setae, 1 campaniform	Rounded	31–41 on distal apex and apicolateral 1/4	Present	Short	This study
	* galesa *	1 seta, 2 campaniform	1 seta	2 setae	2 setae, 1 campaniform	Rounded	50–60 on distal apex, 10–11 lateral ones	Absent	Long	Spinelli et al. (2013)
	* glabra *	1 seta, 2 campaniform	?	?	?	Rounded	50–60 on distal 1/3	?	Long	Wirth (1983a)
	* pulchripes *	1 seta, 2 campaniform	?	?	?	Bilobed	40–52 on distal apex	Absent	Long	Mayer (1959)
	* roldani *	1 seta, 2 campaniform	2 setae	2–3 setae	2 setae	Rounded	50–60 on distal 1/3	Absent	Long	Ronderos et al. (2007); Spinelli and Wirth (1989)
	* snowi *	1 seta, 1 campaniform	?	?	?	Rounded	11–12 on distal 1/4	?	Short	Spinelli and Wirth (1991)
	* ventanensis *	1 seta, 1 campaniform	2 setae	2 setae	1 seta, 1 campaniform	Asymmetrical (outer side curved, inner side straight)	13–15 on distal 1/3	Absent	Short	Spinelli et al. (2012)

In addition, a detailed revision during this study revealed that D-4-T of *B.galesa* is a seta and was erroneously described as campaniform sensillum by Spinelli et al. (2013) and D-5-T and D-8-I of *B.ventanensis* described by Spinelli et al. (2012) as campaniform sensilla are long, a thin seta and a medium-sized seta, respectively.

The pupae of *Bezziachilensis* and the other Neotropical known pupae of *Bezzia* share the features of the generic description given by Borkent (2014). However, we agree on the need of a revision of the genus within a phylogenetic analysis and the redescription of the incompletely described immatures, emphasizing as well the importance of describing immatures for a better knowledge of the genus.

## Supplementary Material

XML Treatment for Bezzia chilensis
